# Isolation and characterization of side population stem cells in articular synovial tissue

**DOI:** 10.1186/1471-2474-9-86

**Published:** 2008-06-12

**Authors:** Takeshi Teramura, Kanji Fukuda, Shinji Kurashimo, Yoshihiko Hosoi, Yoshihisa Miki, Shigeki Asada, Chiaki Hamanishi

**Affiliations:** 1 Institute of Advanced Clinical Medicine, Kinki University School of Medicine, Ohno-Higashi, Osaka-Sayama, Osaka 589-8511, Japan; 2 Department of Orthopedic Surgery, Kinki University School of Medicine, Ohno-Higashi, Osaka-Sayama, Osaka 589-8511, Japan; 3 Kinki University Life Science Research Institute, Ohno-Higashi, Osaka-Sayama, Osaka 589-8511, Japan; 4 Department of Biology Oriented Science and Technology, Kinki University, 930 Nishimitani, Kinokawa, Wakayama 649-6493, Japan

## Abstract

**Background:**

Autologous chondrocyte implantation is an established technique for the repair of degenerated articular cartilage. Recently, the detection of side population (SP) cells, which have the ability to strongly efflux Hoechst 33342 (Ho) fluorescence dye, has attracted attention as a method of stem cell isolation. Although SP cells from synovial tissue were expected to be an excellent source for this tissue engineering, their precise character in the synovial tissue has not been determined.

**Methods:**

Synovial tissues from bovine metacarpophalangeal joints were used as a stem cell source. For efficient collection of stem cells, we first prepared a preculture before sorting in medium containing FBS at variable concentrations for 4 days. Using a cell sorter and the Ho-dye, a poorly stained population enriched with stem cells was then isolated. To determine the characteristics of the stem cells, specific marker genes such as CD34, Flk-1, c-Kit, Abcg-2 were identified by real-time PCR. Sorted SP cells were cultured in a stem cell medium supplemented with bFGF, SCF and fibronectin, and evaluated for their differentiation potentials into chondrocytes, osteocytes and myocytes.

**Results:**

SP cells of synovium tissue were increased from 2% of the total cell population to approximately 10% of the total cells by preculture in the 1%FBS contained medium. Sorted SP cells expressed CD34, Flk-1, c-Kit, Abcg-2 and Mdr-1 -all are important marker genes for stem cell characteristics. The SP cells could be further expanded *ex vivo *while maintaining stem cell potentials such as marker gene expression, Ho-dye efflux potential and multiple differentiation potentials into chondrocyte, osteocyte and myocyte.

**Conclusion:**

In the present study, we demonstrated that the cells with outstanding stem cell properties were efficiently collected as a SP fraction from bovine synovial membrane. Furthermore, we have described an efficient isolation method and the culture conditions for *ex vivo *expansion that maintains their important characteristics. Our results suggest that the SP cells of synovium tissue might be important candidates as sources for cell transplantation.

## Background

Lesions on articular cartilage are difficult to heal spontaneously, and there is no effective strategy for inducing their repair. Autologous chondrocyte implantation (ACI) is now an established technique for the repair of symptomatic isolated lesions of articular cartilage in young adults [1,2]. However, surgical invasion of normal articular cartilage and limited ex vivo expansion of the chondrocytes lead to difficulties in repairing large defects.

Stem cells from synovium membrane have been viewed as fascinating potential sources for regenerative medicine since they possess high chondrogenic properties [3,4] and are possible to be prepared from the joint without damaging the healthy articular cartilage. However, the specific cell markers of these have not yet been determined, and a way for efficient purification also has not been developed.

Recent developments in stem cell biology demonstrated the presence of side population (SP) cells. They are identified by their unique fluorescence-activated cell sorting (FACS) profile, regardless of tissue origin. When separated by a flow cytometer with a UV laser, SP cells are distinct from cells that take up the Hoechst 33342 (Ho) dye [5]. The SP phenotype is determined by the BCRP1/ABCG2 gene that is expressed in pluripotent ES cells and multipotent somatic stem cells [6]. Isolation of SP stem cells by the Ho-dye and FACS technique has been reported in muscular tissue [7,8], liver [9,10], lung [11], skin [12], uterus [13], testis [14] and cornea [15,16] at present.

It was reported that SP stem cells of bone marrow origin displayed strong hematopoietic reconstituting activity as measured by competitive repopulation assays [5], and marrow SP cells also give rise to endothelial cells and skeletal and cardiac muscles [7,8,17]. Moreover, SP cells from the skeletal muscle might be reconstituted as both the skeletal muscle and the hematopoietic system of recipients. From these, it is expected that the SP stem cells of somatic tissues possess superior properties, and it is easy to surmise that SP cells in synovium tissues also possess valuable properties compared to other types of somatic cells, and are expected to be a better source of regenerative medicine.

SP stem cells of synovium tissue have been found by Yamane *et al*., to possess high chondrogenic properties [18]; however, the precise characteristics such as stem cell marker gene expression profiles or multiple differentiation properties were not determined. Furthermore, the culture system for the SP stem cells has not been developed.

In the present study, we determined that the SP cells expressed important stem/progenitor cell markers CD34, Flk-1, c-Kit and Abcg-2, and they possessed multiple differentiation potentials to differentiate into chondrocyte, osteocyte and myocyte. Furthermore, we also succeeded in expansion *in vitro *without losing these properties. Our method suggests a way to amplify necessary stem cells from a little volume of tissue, it might be an effective way to prepare materials for regenerative medicine.

## Methods

### Preparation of synovial cells

Fibrous synovial membrane (wet weight 1.5–2.5 g) was obtained aseptically from the metacarpophalangeal joint of freshly slaughtered calves about 10 months of age, which was kindly gifted from the local slaughterhouse. The specimens were rinsed twice with PBS supplemented with penicillin, streptomycin and amphotericin B, and minced well. They were then digested with 0.1% collagenase (Sigma-Aldrich, St. Louis, MO, USA) in Dulbecco's modified Eagle's medium (DMEM-1; Nissui Pharmaceutical, Tokyo, Japan) containing 10% fetal bovine serum (FBS; Hyclone Laboratories Inc., Logan, UT, USA. Lot no. KPK22095) and Penicillin – Streptomycin – Amphotericin B mixture (Antibiotic-Antimycotic; Invitrogen Corporation, Carlsbad, CA, USA.) for 8 hours at 37°C. Following digestion, the cells were filtered through a 100-μm mesh. The filtrate was centrifuged, washed twice with PBS and stored on ice until further use.

### Hoechst 33342 exclusion assay using fluorescence-activated cell sorting

Released cells were resuspended at a concentration of 10^6 ^cells/ml in FACS buffer composed of DMEM (DMEM-2; Nissui Pharmaceutical) plus 2% FBS, 10 mM HEPES and antibiotics. For preliminary study, we performed Hoechst33342 (Ho; Sigma-Aldrich. Lot no. 017K4122) staining at various concentrations (1, 1.2, 1.4, 1.6, 1.8, 2, 3, 4 and 5 μg/ml) followed by FACS analysis, and confirmed optimal concentrations as 1.8 to 2.0 μg/ml in our system. For analyzing the rates of SP fractions, cell suspensions were incubated in FACS buffer containing Ho-dye at 1.8 μg/ml for 90 minutes at 37°C. For sorting, cells were stained with 2.0 μg/ml under the same conditions to perform subsequent analysis closely. As a negative control for the Ho staining, 50 μM verapamil (Ver; Sigma-Aldrich. Lot no. 096K4617), which blocks the ABC membrane transporter from extruding the Hoechst dye, was added to an aliquot of cell solution. After staining, cells were washed twice with ice-cold FACS buffer, centrifuged and resuspended. Cell sorting was performed using a dual-laser fluorescence-activated cell sorter (FACS VantageSE, Becton Dickinson, San Jose, CA, USA). Ho was excited by an argon multiline UV laser (333.4- to 363.8-nm; COHERENT, Santa Clara, CA, USA.), and fluorescence emission was detected through 450-nm band-pass (Hoechst blue) and 675-nm long-pass (Hoechst red) filters, respectively. Cells showing reduced fluorescence of both blue and red were collected as SP cells. Stained control cells were classified as non-side population (NSP) cells.

### Preculture for efficient collection of SP cells

Digested and filtered cells were cultured on gelatin-coated dishes at 1.5 × 10^6 ^cells/100-mm dish in DMEM or StemPro-34SFM (Invitrogen) supplemented with antibiotics, 0.1 mM pyruvate and 1, 5, 10, 15% FBS. After 4 day's culture, the cells were released from culture dishes with 0.25% trypsin-0.02% EDTA for 3 min, centrifuged and washed with 10% FBS-containing DMEM. The cells were then used for FACS sorting.

### *Ex vivo *expansion of sorted SP cells

Precultured and sorted SP cells were plated onto fibronectin (Fn; BD Falcon, Oxnard, CA, USA) coated 24-well dishes at 10,000 cells per well in Stem Pro-34SFM supplemented with 1%FBS, 10 ng/ml human recombinant basic FGF (bFGF; Upstate, Charlottesville, VA, USA), 25 ng/ml human recombinant stem cell factor (SCF; Peprotech Inc., Rocky Hill, NJ, USA) and insulin-transferrin-serenium (ITS-G; Invitrogen). When 80%–90% confluent, cells were trypsinized and subcultured on Fn coated 35 mm dishes at 5 × 10^4 ^cells per dish. After passaging to 35 mm dish, the culture medium was changed every other day. Passaging was performed every 3 days and continued 12 times.

### *In vitro *differentiations into chondrocytes, osteocytes and myocytes

Precultured and sorted cells were harvested onto fibronectin-coated dishes, and cultured in 1% FBS containing StemPro-34SFM supplemented with 10 ng/ml human recombinant basic FGF (bFGF; Upstate, Charlottesville, VA, USA), 25 ng/ml human recombinant stem cell factor (SCF; Peprotech Inc., Rocky Hill, NJ, USA) and insulin-transferrin-serenium (ITS-G; Invitrogen). To confirm the differentiation potency, we used 7-times passaged cells for the differentiation experiment.

The *in vitro *chondrogenesis assay was performed by pellet culture. 2.0 × 10^5 ^viable SP and NSP cells were centrifuged, and the cells were cultured in DMEM (Nissui Pharmaceutical) supplemented with 5%FCS, ITS, 10 ng/mL transforming growth factor β1 (TGF-β1) (R&D Systems, Abingdon, UK), 10 nM dexamethasone (Sigma-Aldrich), 10 mg/mL ascorbic acid (Sigma-Aldrich), and antibiotics (Invitrogen). After 3 weeks, pellets were fixed with 10% formalin, embedded in tissue-tek compound, sectioned at 7-μm and stained with toluidine-blue and alcian-blue according to standard protocols using cryosections prepared from a bovine articular cartilage as controls [see Additional file 1].

For osteocyte induction, cells were plated at 3 × 10^3^/cm^2 ^in 12-well tissue culture plate and cultured in 10%FBS-DMEM contained 100 nM dexamethasone, 10 mM ascorbic acid, and 2-glycerophasphate. Differentiated samples were tested for alkaline phosphatase (ALP) activity by alkaline phosphatase kit (SIGMA-Aldrich) and calcium deposition by staining with 1% Alizarin red-S solution.

For myocytes differentiation, cells were plated to fibronectin-coated 35-mm dishes and cultured in DMEM high-glucose, 1%FBS, antibiotics and 0.1 mM Pyruvate. After a week, the cultures were fixed, permeabilized by 0.5% Triton-X (SIGMA-Aldrich) diluted in PBS (0.5% PBT), blocked with 5% skim milk (SIGMA-Aldrich) and incubated with anti-DESMIN rabbit IgG polyclonal antibody (Santa Cruz Biotechnology, Santa Cruz, CA, USA, 1:50 dilution) and kept overnight at 4°C. For immunofluorescence microscopy, the samples were incubated for 1 h at room temperature with FITC-conjugated bovine anti-rabbit IgG antibody (Santa Cruz Biotechnology, 1:1000 dilution). These samples were counterstained with propidium iodide (PI) before observation.

### RNA extraction and real-time PCR analysis

Sorted cells and cultured cells were treated with TRIzol reagent (Invitrogen) and mixed thoroughly with pipetting. To dissociate the pellets of chondrocyte obtained by differentiation assay, the pellet was disrupted using a 21-G needle and syringe in TRIzol reagent. The Trizol-lysates were then mixed with chloroform and centrifuged at 15,000 × g for 15 minutes. Following centrifugation, total RNA were obtained by isopropanol precipitation according to the manufacturer's instructions. Single strand cDNA was prepared from total RNA using Random primer under standard conditions with the High Capacity cDNA reverse transcription kit (Applied Biosystems, Foster City, CA, USA). Quantitative real-time PCR with total cDNA was performed using Perfect real-time SYBR green (Takara Bio, Inc., Shiga, Japan). PCR amplifications were performed with the 7700 real-time PCR System (Applied Biosystems) at 95°C for 10 sec followed by 40 cycles of 95°C for 5 s, 60°C for 30 s. To quantify the relative expression of each gene, the Ct (threshold cycle) values were normalized for endogenous reference (delta Ct = Ct target- Ct β actin) and compared with a calibrator, using the "delta-delta Ct method" (delta-delta Ct = delta Ct_sample _- delta Ct_calibrator_) [19]. As a calibrator, we used the average Ct value of synovium tissues obtained from three different animals. Using the delta-delta Ct value, relative expression was calculated (2^-deltadeltaCt^). As the delta Ct method is only applicable when the amplification efficiencies of the target and the reference are essentially equal, we determined the efficiencies for 5 dilutions, and the delta Ct values (Ct_target_- Ct_β actin_) were plotted against the dilution (log). The slope of the fitted line was then determined. A slope of less than 0.1 is then indicative of equal efficiencies (data not shown). The target genes in the present experiment were CD34, Flk-1, c-Kit, Abcg-2 and Mdr-1. All experiments included negative controls consisting of no cDNA for each primer pair. All samples were tested in duplicate, and the average values were used for quantification. Primers were designed to span exons to distinguish cDNA from genomic DNA products (Table 1).

**Table 1:** Primers using the characteristics of SP cells and differentiated cells

	**NCBI Gene ID**	**Forward**	**Reverse**	**Amplicon**
*β-actin*	280979	AGGTCATCACCATCGGCAAT; ex 3*	GAATGCCGCAGGATTCCAT; ex 4	87 bp
*CD34*	281051	TGCTATTTCCTGATGAACCGC; ex 7	TCCACGTAATAAGGGTCTTCGC; ex 8	74 bp
*Flk-1*	407170	TTCCAAGTGGCTAAGGGCAT; ex 22	TTTAACCACGTTCTTTTCCGACA; ex 23	102 bp
*c-Kit*	280832	CAAGGAAGGTTTCCGAATGC; ex 19	CCCAGCAGGTCTTCATGATGT; ex 20	74 bp
*Abcb-1 (Mdr-1)*	281585	GCAAAGCAGGCGAGATCC; ex 17	TCAATGCTCCCGTGGTGTT; ex 18	111 bp
*Abcg-2 (Bcrp-1)*	536203	CCTTGGTTGTCATGGCTTCA; ex 14	AGTCCTGGGCAGAAGTTTTGTC; ex 15	98 bp
*Pcgf-4 (Bmi-1)*	510666	GGCTGGAACCGCCTAAAAC; ex 1	TCTGCTTGATAAAAGATCGGCTC; ex 1–2	91 bp
*COL1A1*	282187	ATGGCGAAGCTGGAAAG; ex 9–10	CCACTGAAACCTCTGTGT; ex 11–12	121 bp
*COL2A1*	282189	TGGTATCGCCGGACCCAAG**	CTCGTCCACCGTCCTTCCC**	82 bp
*Aggrecan*	280985	CACCTGTAAAAAGGGCACAGTG; ex 15	GCATTGATCTCGTATCGGTCC; ex 16	95 bp
*CD-RAP*	280857	TGACCGGAAGATGTGTGCC; ex 2	CACGTAGTCCTGAAGGGCCA; ex 3	73 bp
*MyoD-1st*	281938	CAAACGCAAGACGACTAACG; ex 1	TGTAGTAAGTGCGGTCGTAG; ex 2	392 bp
*MyoD-2nd*	281938	AACGCCATCCGCTATATCG; ex 1	TGTAGTCCATCATGCCGTCG; ex 2	187 bp
*Myf-5*	281335	ACATTGAGAGTCTGCAGGAG; ex 1	TTGCTCTGAGTTGGTGATC; ex 3	269 bp
*Osteocalcin*	281646	AGATGCAAAGCCTGGTGATG; ex 2	ATGTGGTCAGCTAGCTCGTC; ex 4	192 bp

*Number of exons (ex) to which the primer binds.

**Detailed information has not been reported.

### RT-PCR analysis for evaluation of differentiation

The cDNA from each sample was diluted and used for an RT-PCR-based assay for type I collagen (COL1A1), type II collagen (COL2A1), CD-RAP, Aggrecan, Myo-D, Myf-5 and Osteocalcin. PCR amplifications were performed at 95°C for 2 min followed by 35 cycles of 94°C, 20 s; 58°C, 20 s; and 72°C, 20 s using Platinum Taq PCRx DNA polymerase (Invitrogen) with appropriate primers according to the manufacturer's instructions. All primers for PCR were designed on two different putative exons so as to span one intron or designed to span a putative exon-exon junction. Amplicons were analyzed by agarose-gel electro-phoresis and ethidium bromide-staining.

### Statistical analysis of the data

Significant difference was detected by one-way ANOVA analysis followed by Tukey's multiple comparison tests. Comparisons between cultured SP cells and cultured NSP cells were performed by student t-test, and gene expression alteration after passages of SP cells were evaluated by Dunnet's test using the values of the SP cells at primary culture as controls. A P-value of less than 0.05 was considered significant.

## Results

### Characterization of SP cells in synovial tissue

**Figure 1:**
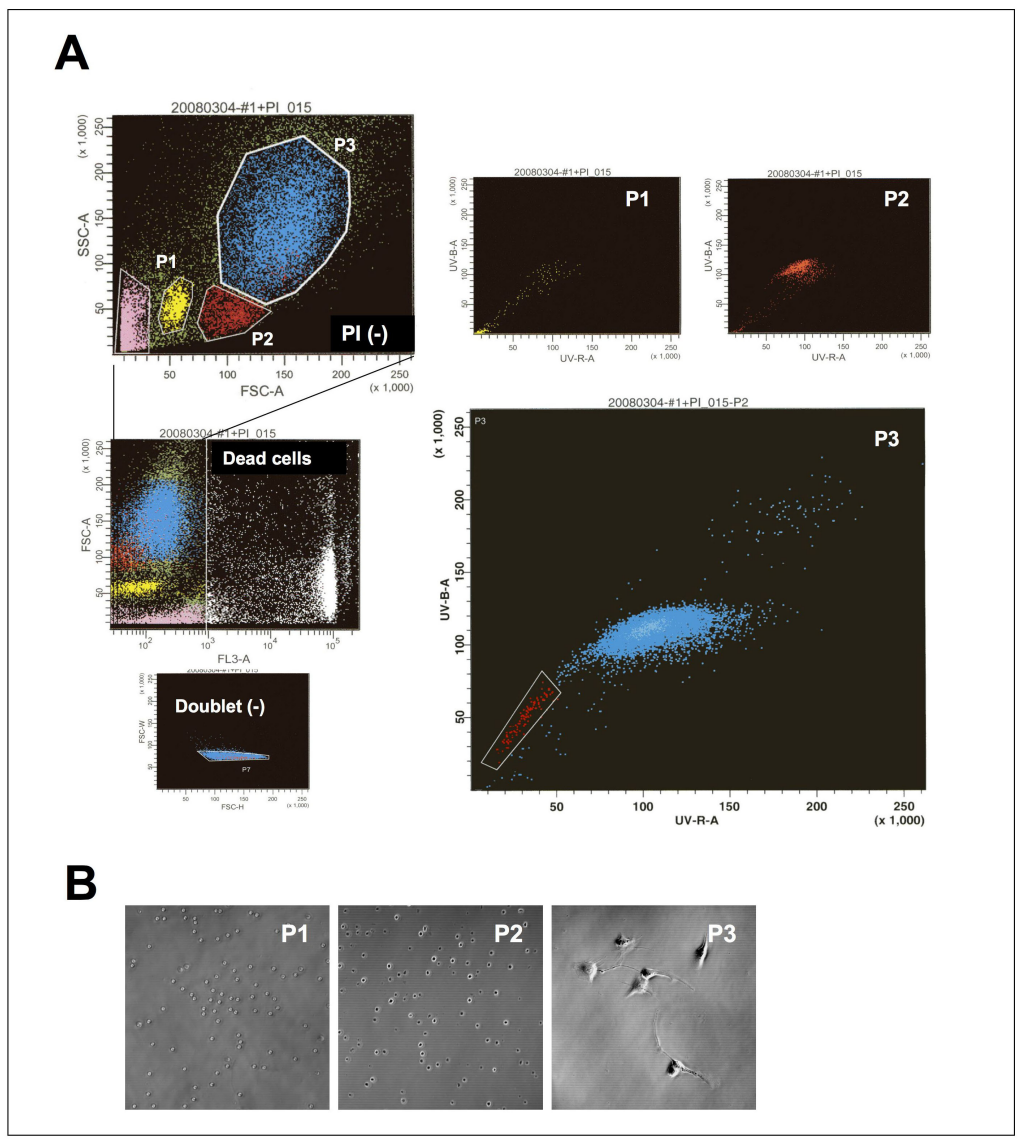
**Flow cytometric characterization of SP cells**. (A) Bovine synovial cells were stained with Hoechst 33342, and excited by a UV laser. Populations including SP cells were determined by live/dead evaluation using PI, single/doublet cells evaluation using FSC-A/SSC-A and microscopic evaluation (B). SP patterns were visible after Hoechst 33342 staining in population 3 (P3). P1 and P2 consisted of small, non-adherent cells.

Bovine synovial cells were stained with the vital DNA dye, Hoechst 33342, and excited by a UV laser. Usually, the SP cells exhibit a low blue (440- to 460-nm) and low red (>675-nm) fluorescent staining pattern. This pattern is created by an efflux of the Hoechst 33342 dye from the SP cells. In case of the bovine synovial cells, three distinct regions were observed after excluding dead cells and debris. The cells included in population 1 (P1) and population 2 (P2) were smaller than that of population 3 (P3), and no adherent cells were observed when they were seeded on culture dishes. On the other hand, population 3 consisted of fibroblast-like cells and included a distinct side population (SP) (Figure 1). The dye exclusion property of SP cells is partly due to the functional ABC transporters and verapamil effectively blocking the ABC membrane transporter from extruding the Hoechst dye [20-24]. With the addition of verapamil, the presence of the SP fraction in region 3 was clearly eliminated [see Additional file 2], indicating that the cells in this region were candidates for stem cells. The SP cells expressed stem cell maker genes such as CD34, Flk-1, c-Kit, Abcg-2 and Mdr-1.

### Preculture for expansion of SP cell

**Figure 2:**
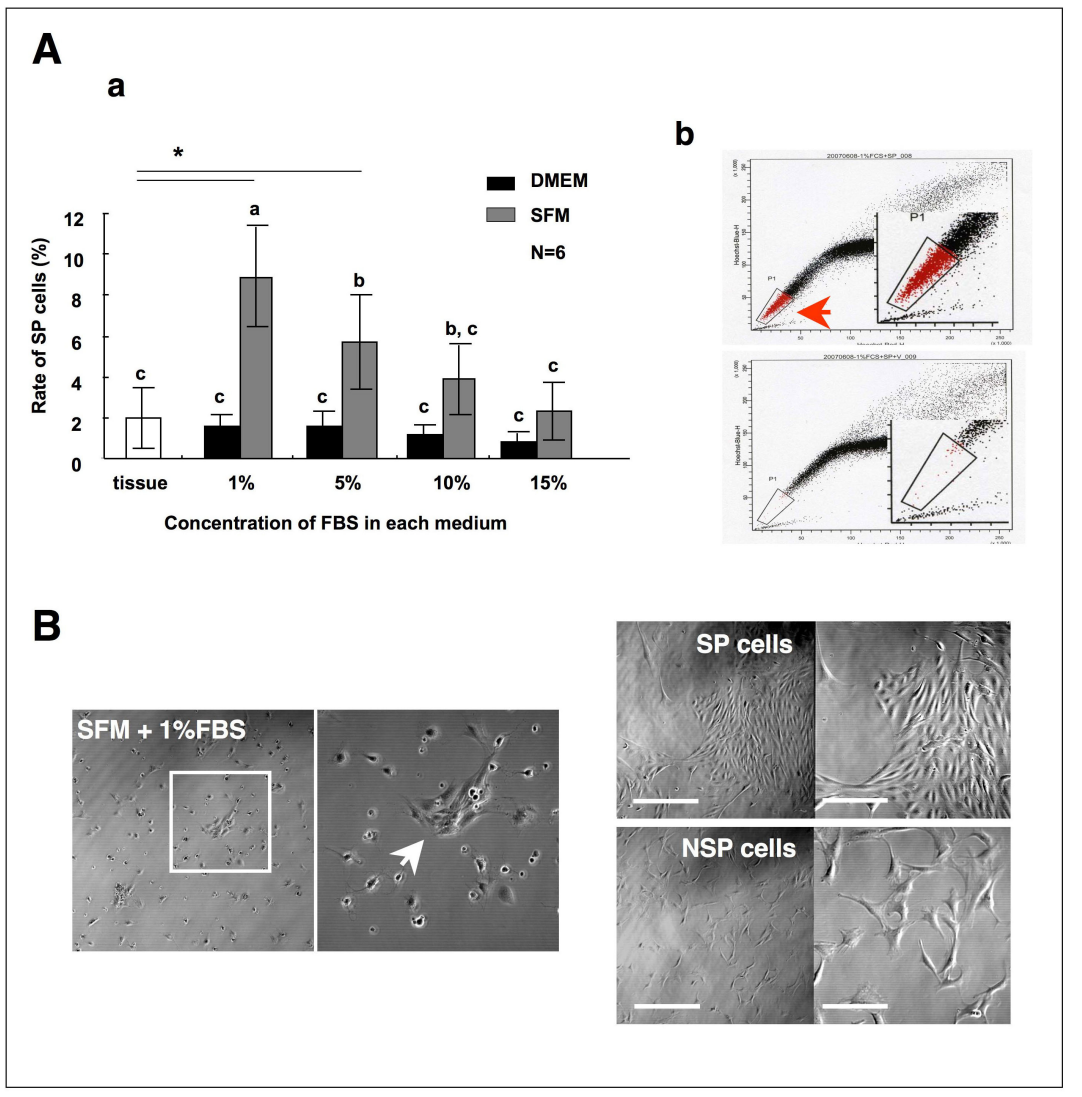
**Expansion of SP cells by preculture**. (A-a) Synovial tissue-derived SP cells were cultured in the DMEM (solid rectangles) or StemPro-34SFM (SFM; shaded rectangles) with different concentration of FBS. Data represent the mean ± S.D. of 6 experiments. Asterisks indicate statistically significant difference (P < 0.01) between control (tissue) evaluated by Dunnet's test. Different characters indicate statistically significant difference (P < 0.05) determined by ANOVA and Tukey's multiple comparison tests. (A-b) SP region expanded by preculture in 1%FBS supplemented SFM (upper panel). The regions were determined by verapamil treatment (lower panel). (B) Phase-contrast images of precultured cells in SFM supplemented with 1% FBS. In the condition, small cell colonies could be observed (arrow). Phase-contrast images of the sorted cells (right). Right-upper panels show SP cells (scale bar = 200 μm) and high-magnification image of these (scale bar = 100 μm). Right-lower panels show NSP cells and high-magnification images of these.

From the gene expression profiles, SP cells in P3 might be stem cells in synovial tissues. However we found that the proportion of SP cells was less than 2%, and it was clear that cell number enhancement is necessary to obtain the cells efficiently. We examined several culture conditions to amplify SP cells. This procedure was also reported to be effective for collecting SP cells from a small amount of tissues, such as corneal stroma [15]. When synovial tissue-derived cells were cultured in ordinary medium (DMEM containing 10% FBS), only fibroblast-like cells with larger cytoplasm grew rapidly, while the rate of SP cells did not increase (Figure 2). In the culture using DMEM, altering the FBS concentration did not affect the rate of SP cells. Then, we used stem cell specific medium StemPro-34SFM. Unexpectedly, synovial tissue-derived cells neither adhered to the culture plate nor multiplied under serum-free conditions [see Additional file 3]. The result suggested that additions of growth factors and/or cell adhesion molecules contained in serum are essential to the survival of synovial tissue derived cells. Accordingly, we tested serum addition to the medium at various concentrations. The rate of cell adhesion and growth was significantly enhanced when serum was added at 1 to 5% concentrations to the medium.

In contrast, less than 1% serum addition could not support cell adhesion and proliferation, and over 10% serum addition significantly diminished the expansion of SP cells. In 1% FBS containing StemPro-34SFM, small spindle-like cells were expanded, and formations of small colonies were observed. Therefore, we determined the ideal culture condition for the synovial SP cells to be 1% FBS containing StemPro-34SFM.

### Characteristics of SP cells

**Figure 3:**
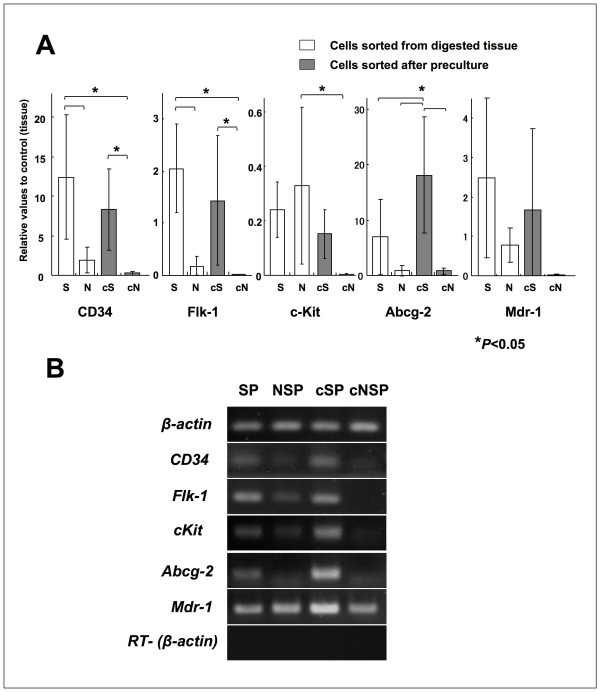
**Analysis of the stem cell marker gene expressions based on quantitative RT-PCR**. (A) Expression scores were obtained by the delta-delta Ct calculation method. All values are mean ± S.D. of 5 samples of SP (S) and NSP (N), and 6 samples of precultured SP (cS) and precultured NSP (cN). Significant differences were observed using ANOVA and Tukey's multiple comparison tests. Asterisks on bars indicate significant differences (P < 0.05). (B) Gel electrophoresis pattern recognition of real-time PCR products. Total RNA without reverse transcribed samples (RT-) were used as negative control.

After preculture in 1% FBS containing StemPro-34SFM, we examined the characterization of cultured SP cells for the stem cell markers. The SP cells showed significantly higher expression, than NSP cells, of somatic stem cell marker genes CD34, Flk-1, and Abcg-2 (Figure 3).

### Culturing and analysis of SP cells

**Figure 4:**
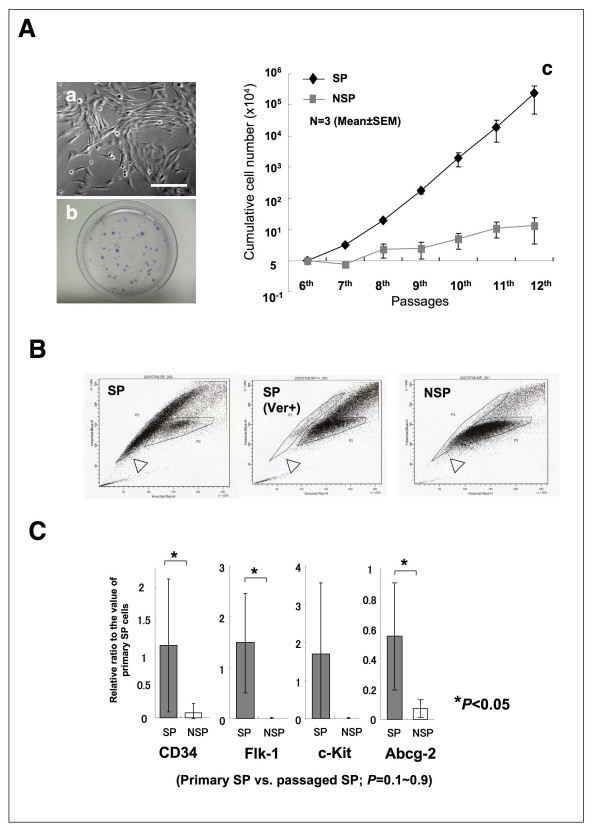
**Characteristics of SP cells on *ex vivo *expansion**. (A-a) Phase-contrast images of cultured SP cells. Scale bar = 100 μm. (A-b) Colony-forming assay with the SP cells. SP cells were seeded at a density of 500 cells per a fibronectin coated 35-mm dish. (A-c) Cell proliferation speed was markedly promoted by addition of growth factors and fibronectin addition. Values are the mean and SEM of 3 independent cell lines. (B) Ho-staining and FACS analysis of *ex vivo *expanded SP and NSP cells. Open triangles indicate the populations possessing Ho-efflux properties. The NSP cells did not represent the Ho-efflux property. All experiments were performed using 6 times passaged cells, and replicated 3 times using 3 independent cell lines. (C) Analysis of the stem cell marker gene expressions based on quantitative RT-PCR in *ex vivo *expanded SP cells. Y-axis represents the relative values for SP cells of primary culture (correspond to the mean values of cS in figure 3A). Significant differences between primary SP cells and 7 times passaged SP cells were observed by Dunnet's test. Significant differences between passaged SP cells and passaged NSP cells were observed by student t-test.

To stably supply transplantable cells, *ex vivo *amplification of the cells was required. Because we showed that SP cells in the synovial tissues could be amplified using precultured medium, *i.e*. SFM and 1% serum, sorted SP cells were cultured under the same condition; however, the SP cells after sorting did not adhere and proliferate. It is possible that, in primary culture, co-existence of variable types of cells including blood cells secret some cytokines or cell adhesion molecules in an autocrine or paracrine fashion, and support the adhesion and proliferation of SP cells. Next, we examined the effect of bFGF, SCF and fibronectin on the adhesion and growth of the SP cells. Sorted SP cells exhibited small spindle-like features and formed colonies when cultured in the condition with these supplementations, on the other hand, NSP cells did not form colonies, and the proliferation speed was low. In the culture condition, the SP cells exhibited logarithmic proliferation even after 10 passages and Ho-efflux properties were also maintained. Furthermore, the SP cells expressed marker genes CD34, Flk-1, c-Kit and Abcg-2 (Figure 4).

### Determination of multiple differentiation potentials

To confirm the multilineage differentiation potential, SP cells, which were expanded *ex vivo *and passaged more than 7 times, were induced to differentiate into chondrocyte, osteocyte and myocyte. In chondrogenic culture, the cell pellets became spherical, and the pellets from SP cells tended to have greater amounts of cartilage matrix than did pellets from NSP cells as shown by staining with toluidine blue and alcian blue. RT-PCR analysis demonstrated further characterization of chondrogenesis, and chondrocyte specific genes COL2A1, CD-RAP and Aggrecan were determined.

**Figure 5:**
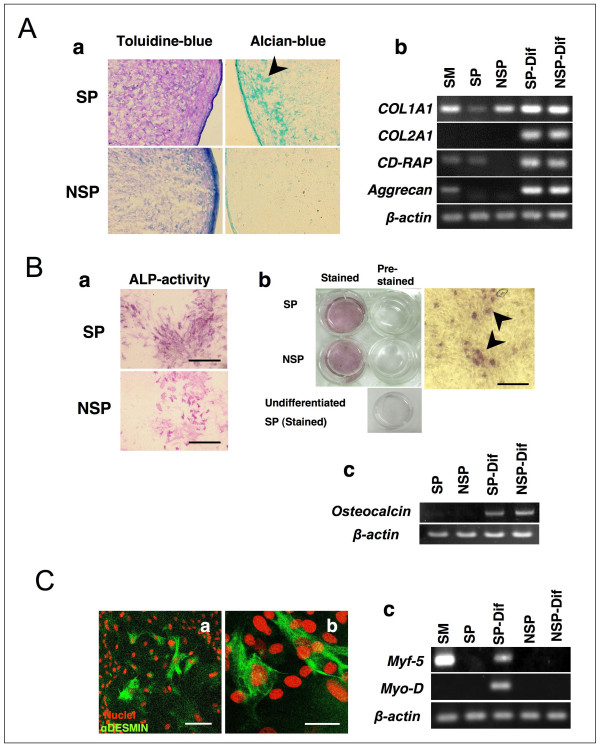
**Evaluation of differentiation properties of SP cells**. (A) *In vitro *chondrogenesis. SP and NSP cells were cultured for 21 days in TGF-β contained chondrocyte differentiation medium. (A-a) Pellets were stained with toluidine blue and alcian blue. (A-b) Expression of chondrogenic genes was also examined. SP and NSP cells were cultured for 21 days, and chondrocyte specific gene expressions were determined by RT-PCR analysis. Representative data were shown with 3 experiments. (B) *In vitro *osteogenesis. SP and NSP cells were cultured for 21 days in osteocyte differentiation medium. (B-a) ALP activity was determined in both derivatives. (B-b) Calcium deposition was examined by 1% Alizarin red staining, High-magnification image of stained cells derived from SP cells were shown in right panel. Scale bar is 200 μm. (B-c) Expression of osteocyte specific gene Osteocalcin was determined by RT-PCR. (C) *In vitro *myogenesis. SP and NSP cells were cultured for 7 days in myocyte differentiation medium. Desmin expressions were determined by immunocytochemical staining (C-a; scale bar = 100 μm), and high-magnification image of SP derived myocyte (C-b; scale bar = 50 μm). (C-c) Myocyte specific gene expressions were determined by RT-PCR.

SP cells also had differentiation potentials to become osteocyte and myocyte. About the osteogenic potentials, no remarkable difference was observed between the origins, *i.e*. SP cells and NSP cells. On the other hand, myogenic property was only observed in SP cells. Desmin expressing myocytes were detected in the derivatives of SP cells, and these cells expressed other myogenic marker genes Myf-5 and Myo-D (Figure 5).

## Discussion

In the present study, we determined stem cell specific marker gene expression of SP cells in bovine synovial membrane, and constructed a culture system for efficient collection and *ex vivo *expansion of the stem cells. Recently, Yamane *et al*. reported that the SP fraction was only 0.6 % in the synovium tissues at the medial and lateral femoral condyles of 3-month-old calves [18]. On the other hand, we observed SP fractions in about 2 % of the total cells. Furthermore, the flow cytometric profile of the two reports was totally different. This discrepancy might be due to differences of tissue origins, it is possible that the numbers of SP cells were different in the area of synovium tissues. Another factor might be based on the difference in the cell sorting condition. For instance, they used 10 μg/ml of Hoechst 33342 dye and we used it at 1.8 – 2.0 μg/ml. The experiment for determination of SP cells was dependent on the staining protocol which is subject to variations in timing, temperature, cell concentration, and Ho-dye concentration. We first determined the gene expression profile of bovine synovium SP cells using some stem cell markers such as CD34, c-Kit, Flk-1, Abcg-2 and Mdr-1. CD34 is a transmembrane sialoglycoprotein expressed by early hematopoietic stem cells [25]. Flk-1 is a receptor for vascular endothelial growth factor, and a marker for progenitor cells of hematopietic and endothelial lineage [26]. C-Kit, also known as CD117, is a receptor for stem cell factor and is expressed in various stem cells and hematopoietic progenitor cells [27,28]. Abcg-2 is the molecular determinant for the SP phenotype and has been postulated as a universal stem cell marker [29]. These gene expressions were observed in some somatic stem cells, and it is thought these are important for stem cell characteristics mainly to maintain their stem-ness. The synovium SP cells also expressed these markers and there was also additional evidence for stem cells.

Although the stem cells collected as SP cells possessed valuable characteristics for stem cell based therapy, the important factor in tissue engineering is to harvest the greatest number of cells with the highest potential while minimizing the amount of mesenchymal tissue needed, for less-invasive treatments. In this context, clearly the most important contribution of our study is the finding that the SP cells could be expanded *ex vivo *under specific conditions. We found that a combination of 1% FBS and StemPro-34SFM is ideal for enhancing SP cell proliferation. Under this condition, the rates of SP fractions were up to over 8% of total cells. Interestingly, increased supplementation of serum to over 10% inhibited the proliferation of the SP cells [see Additional file 4]. It is possible that the high serum concentration induced SP cells to differentiate into somatic cells and promoted expansion of differentiated cells, which caused the relative rate of SP fractions to decrease.

On the other hand, under the conditions of less than 1%, adherent cells were decreased and getting enough cells for cell sorting was difficult. When SP cells were cultured in StemPro-34SFM with 1% FBS, the expressions of stem cell markers were maintained. When ordinal culture medium DMEM was used as basement medium, effects for expansion and decreasing the rates of SP fractions dependent on serum concentrations were not observed. StemPro34-SFM is a medium specifically developed for CD34 expressing hematopoietic stem cell culture and the effects for other types of stem cell such as spermatogonial stem cells were reported [30-32]. Although the mechanism or molecules by which the expansion of SP cells were stimulated in the medium is not known, the factors necessary for the maintenance of these stem cells are the defined components of StemPro34-SFM, and may be shared with hematopoietic stem cells.

To achieve further enhancement of the cell number, we then examined the conditions for *ex vivo *expansion of sorted SP cells. For maintaining the SP cell proliferation while keeping their specific characters as stem cells, we found that the supplementation with three factors (bFGF, SCF and Fn) was effective. The FGF families are important factors for proliferation of variable stem cells such as mesenchymal stem cell (MSC) [33,34], neural stem cell [35], and embryonic stem cell [36,37]. SCF plays an important role via a c-Kit receptor in the recruitment of adult hematopoietic stem cells [27], or formation of germ stem cells *in vivo *[28]. Also *in vitro*, SCF is an essential factor for the maintenance of hematopoietic stem cell [38-40]. Fn is a kind of extra-cellular matrix glycoprotein, which functions in promoting cell adhesion. In hematopoietic tissues, stem cells adhere to the Fn and endothelial vascular cell adhesion molecule (V-CAM) by integrin and cell surface glycosaminoglycans [41]. And the molecule functions as the crucial factor for maintenance of *ex vivo *culture [41,42]. The above culture condition enables the logarithmic expansion while maintaining some important original characteristics. After continued passaging and culturing, the expression of CD34, Flk-1 and Abcg-2 mRNA was still maintained at a high-rate compared with NSP cells. Importantly, this Abcg-2 mRNA expression reflected as the phenotype that the cultured SP cells also represented the Ho-efflux properties and Ver-sensitivity. However, the FACS profiles were little different from the original profiles of SP fractions. It was supposed that the valance of the two kinds of Ho-efflux proteins Abcg-2 and Mdr-1 were altered from original cells. This profile was observed only in SP cell derivatives, and it was lost with differentiation (data not shown).

Recently, it was reported that cells expressing CD34 and CD117 (c-Kit) exist at <2%, and cells expressing Flk-1 exist at <4%, in synovial tissues [4]. Although the expression of these molecules was an important phenotype of stem cells, the expression of Flk-1 was diminished by *ex vivo *culture when cultured in normal medium [43]. The SP cells cultured in our system maintained other markers CD34, Flk-1 and c-Kit expressions. From these, our system might be suitable for maintaining undifferentiated stem cells in synovium tissues.

Furthermore, we also demonstrated multiple differentiation potentials for synovial SP cells into chondrocyte, osteocyte and myocyte. In our study, NSP cells used as control also possessed differentiation properties to differentiate into chondrocyte and osteocyte. It was hypothesized that NSP fractions might include some kind of progenitor cells that could differentiate into chondrocyte or osteocyte. On the other hand, myogenic properties were only observed in SP cells. As stimulating chemicals for myogenic differentiation from somatic stem cells such as MSC *in vitro*, 5' azacytidine that is an analog of cytidine, were used [43,44]. The chemical functions by incorporation into DNA, and results in hypomethylation of the DNA. On the other hand, transplantation to muscular tissues also induces myocyte differentiation from MSCs derived from synovium tissues [45]. However, in our study, the SP cells could differentiate to myocyte without the chemical or transplantation, about 1% of derivatives expressed Desmin, a muscle-specific intermediate filament protein. These results mean that the myogenesis from SP cells might occur as the "forward" differentiation, and suggested that the SP cells maintained high differentiation potentials.

## Conclusion

We first demonstrated the capacity of SP cells in the synovial tissue to proliferate *ex vivo *and differentiate into chondrocytes, osteocytes and myocytes. Although further studies are required to demonstrate that SP cells are transplantable stem cells after *ex vivo *expansion, the present findings might lead to the development of an effective method for preparing materials for cell-based therapy.

## Abbreviations

SP: side population; NSP: Non-Side Population; Abcg-2: ATP-binding cassette subfamily G member-2; MDR-1: Multi Drug Resistance-1; Flk-1: Fetal liver kinase 1; Ho: Hoechst 33342; Ver: verapamil; FBS: fatal bovine serum; DMEM: Dulbecco's modified Eagle's medium; SFM: Stem Pro-34SFM; P1: Population 1; FACS: fluorescence activated cell sorter; Ct: cycle threshold.

## Competing interests

The authors declare that they have no competing interests.

## Authors' contributions

TT carried out the cell culture, molecular genetic studies, immunocytochemical assays and drafted the manuscript. KF carried out the design of the study and prepared the manuscript. SK participated in the cell sorting. YH participated in the design of the study and performed the statistical analysis. YM carried out the tissue preparing and cell culture. SA participated in the preparation of the materials. CH conceived of the study, and participated in its design and coordination and helped to draft the manuscript.

## Pre-publication history

The pre-publication history for this paper can be accessed here:



## Supplementary Material

Additional file 1 Histology of bovine articular cartilage. Cryosections were stained with toluidine blue (left panel) and alcian blue (right panel). These figures are shown as positive controls for histological evaluation of chondrocyte differentiation assay. Click here for file

Additional file 2 Optimization of Hoechst33342 (Ho) dye concentration for detection of side population cells in bovine synovial tissues. Click here for file

Additional file 3 Microscopic images of the synovial tissue derived cells after preculture in various conditions. Click here for file

Additional file 4 FACS profiles of the cells cultured in 1%FBS supplemented SFM (left panel) and the cells cultured in 15% FBS supplemented SFM (right panel). Click here for file
