# Supplementary figures and images for: Isolation and characterization of side population stem cells in articular synovial tissue
Source: BMC Musculoskelet Disord. 2008 Jun 12;9:86. doi: 10.1186/1471-2474-9-86 (PMC2440379; doi:10.1186/1471-2474-9-86)

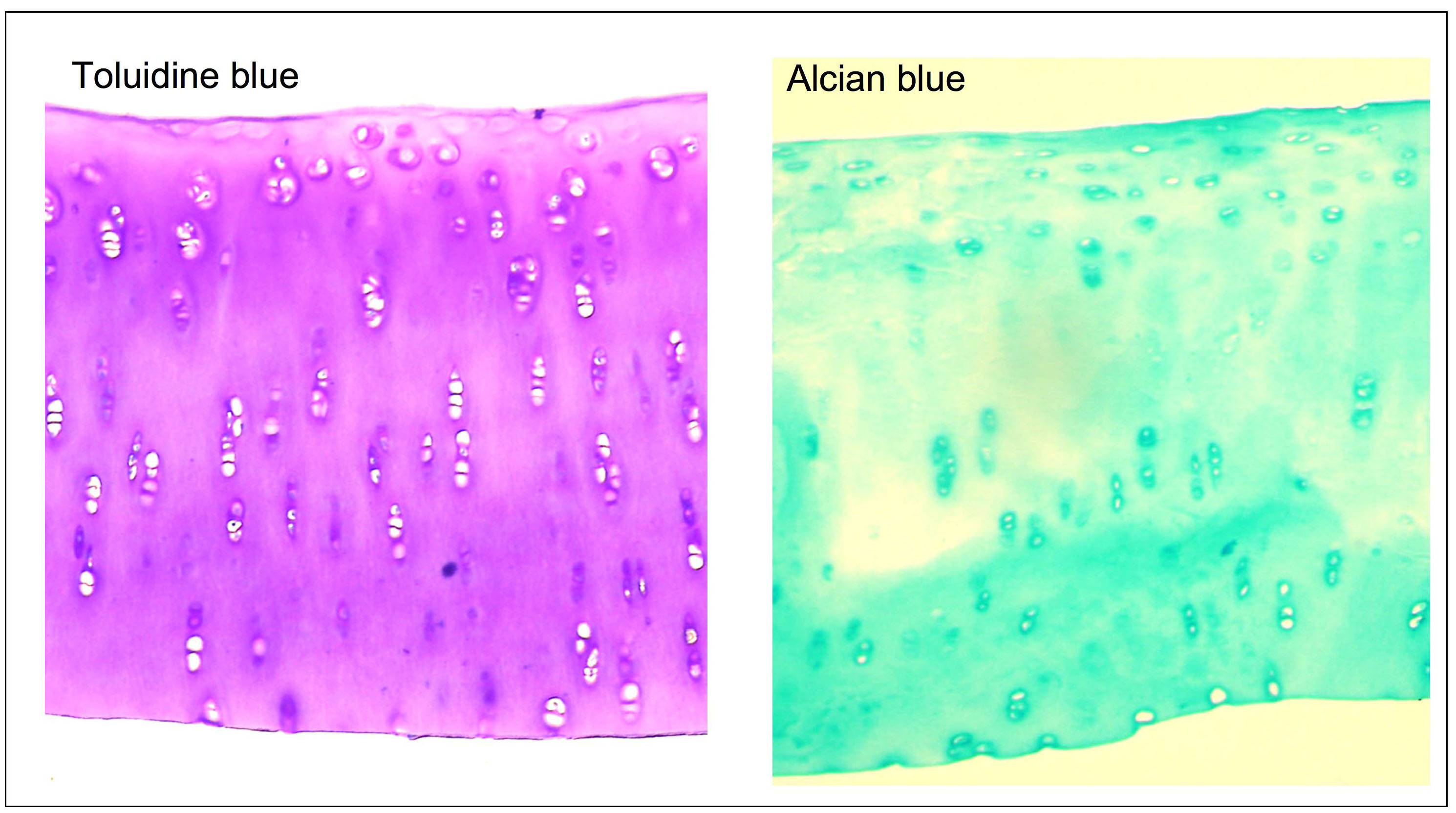

Supplement: Additional file 1 — Histology of bovine articular cartilage. Cryosections were stained with toluidine blue (left panel) and alcian blue (right panel). These figures are shown as positive controls for histological evaluation of chondrocyte differentiation assay. [file 1471-2474-9-86-S1.jpeg]

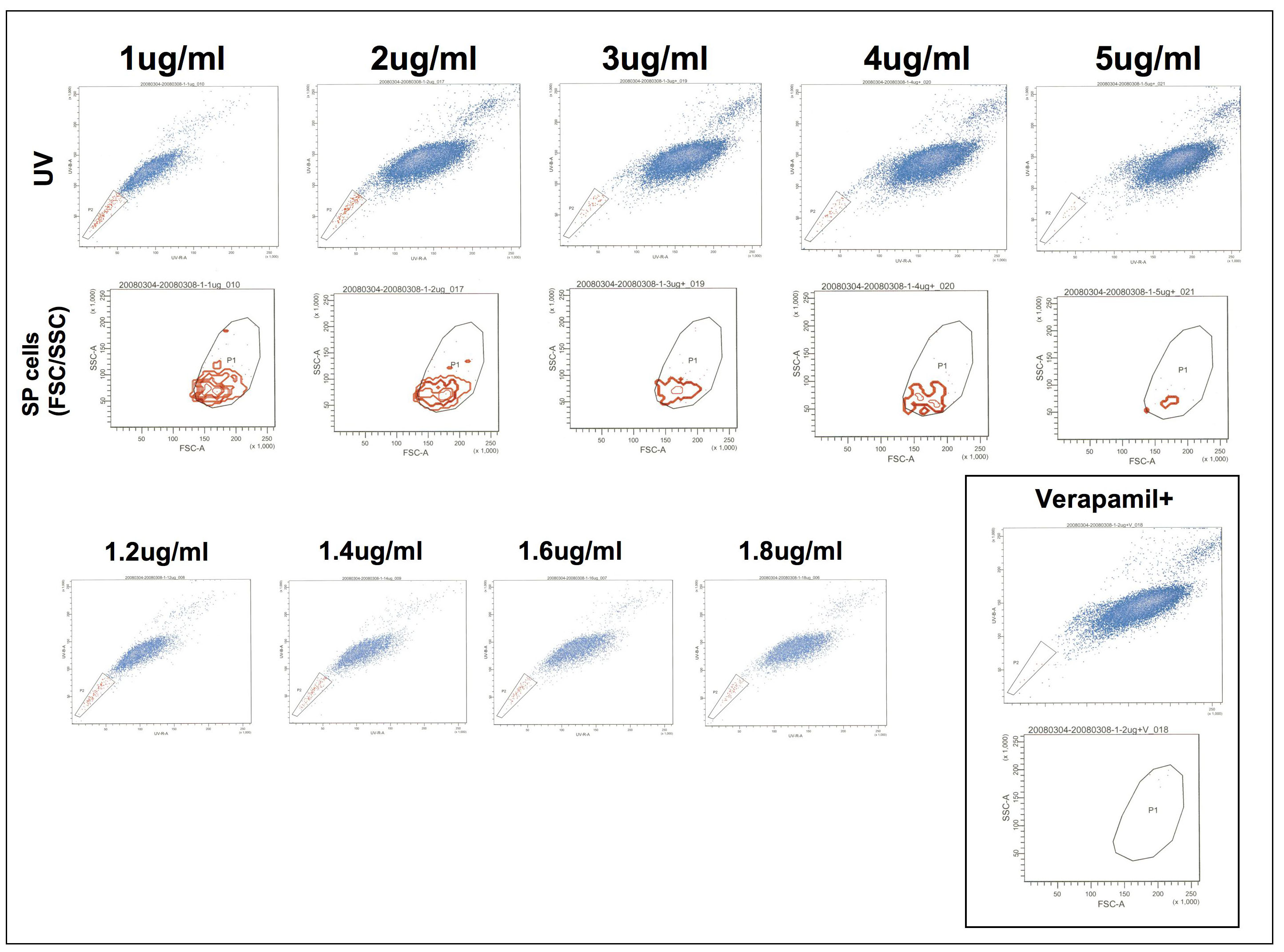

Supplement: Additional file 2 — Optimization of Hoechst33342 (Ho) dye concentration for detection of side population cells in bovine synovial tissues. [file 1471-2474-9-86-S2.jpeg]

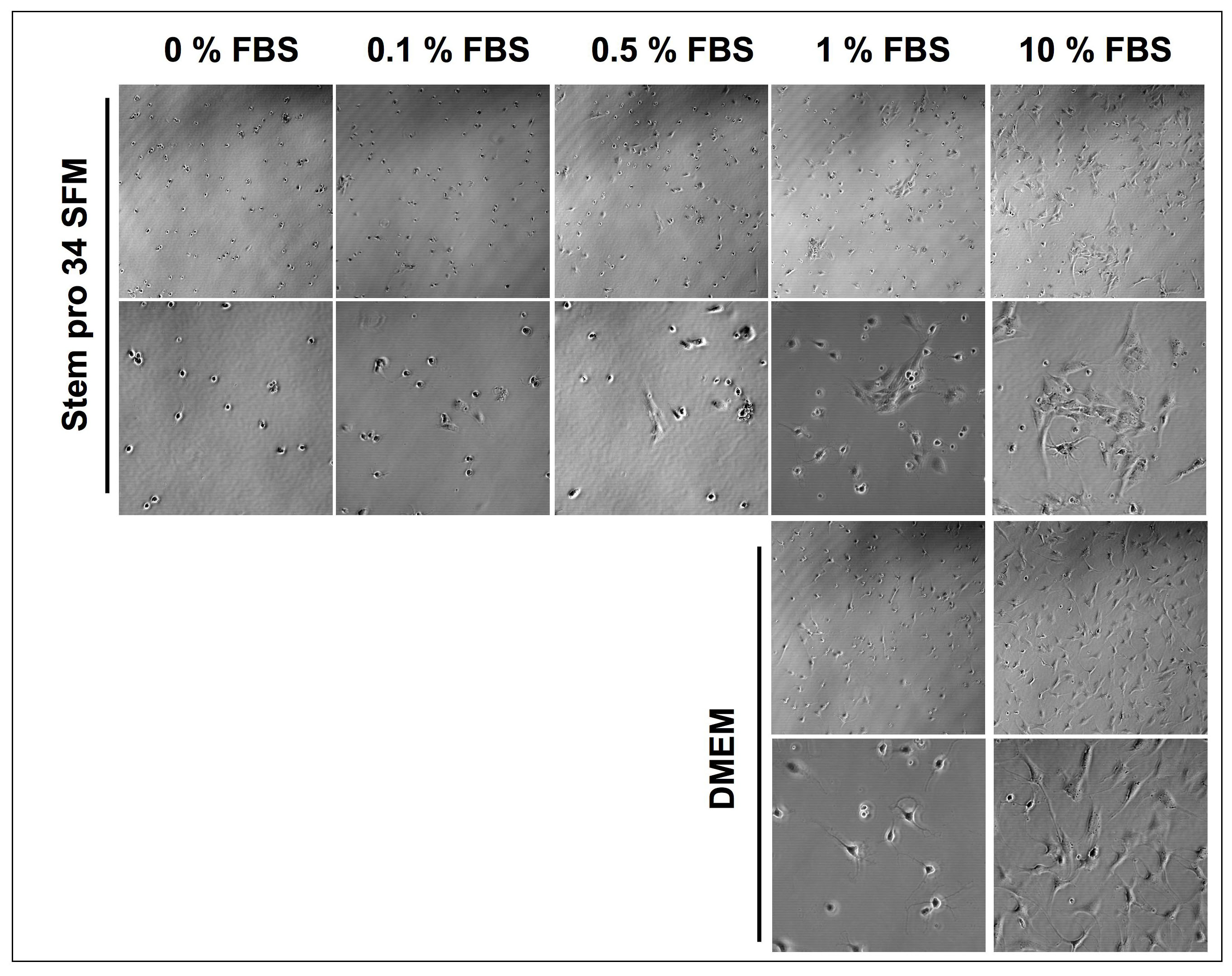

Supplement: Additional file 3 — Microscopic images of the synovial tissue derived cells after preculture in various conditions. [file 1471-2474-9-86-S3.jpeg]

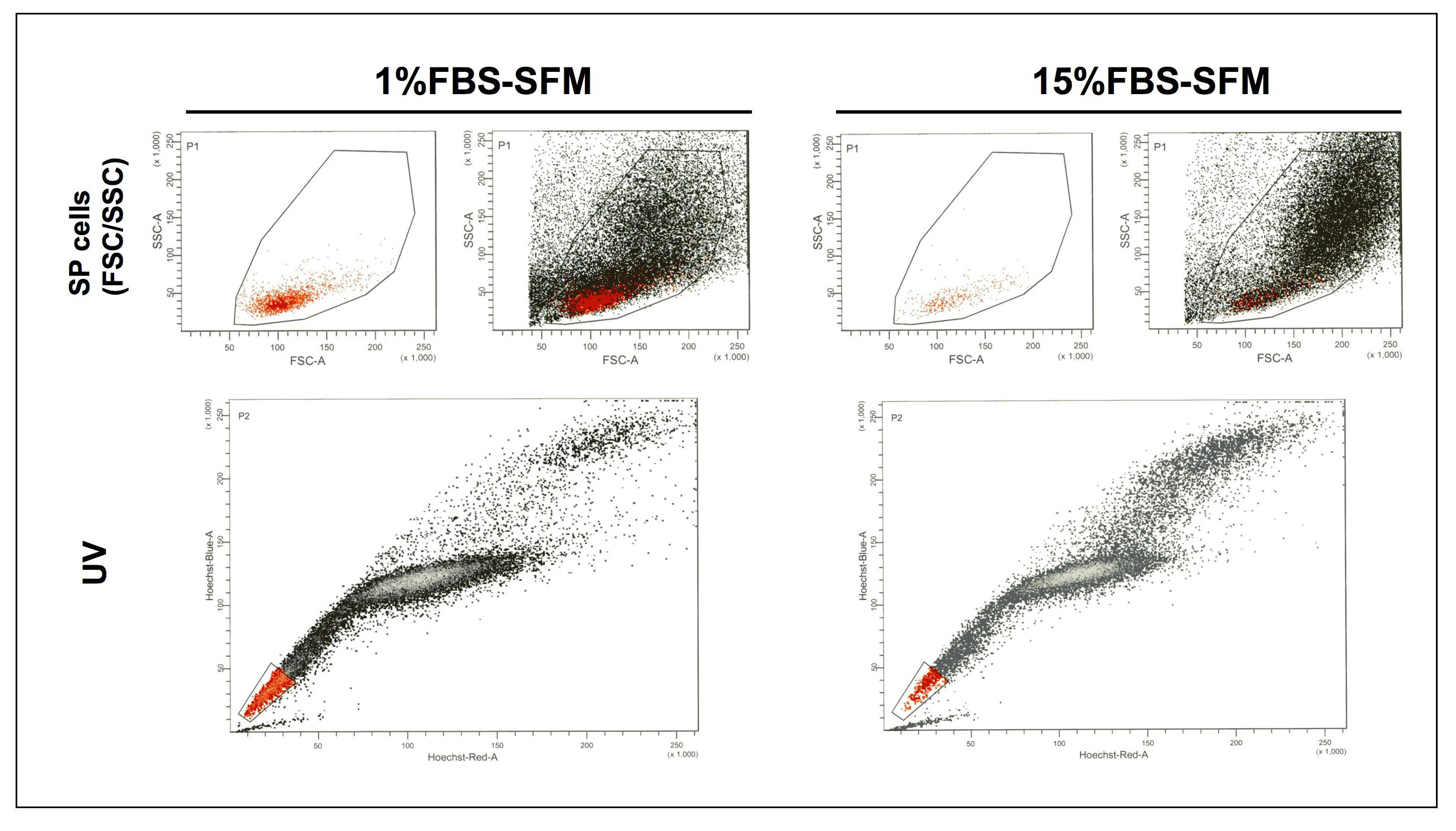

Supplement: Additional file 4 — FACS profiles of the cells cultured in 1%FBS supplemented SFM (left panel) and the cells cultured in 15% FBS supplemented SFM (right panel). [file 1471-2474-9-86-S4.jpeg]
